# Multiplex quantitative PCR for detection of lower respiratory tract infection and meningitis caused by *Streptococcus pneumoniae*, *Haemophilus influenzae *and *Neisseria meningitidis*

**DOI:** 10.1186/1471-2180-10-310

**Published:** 2010-12-03

**Authors:** Guma MK Abdeldaim, Kristoffer Strålin, Jens Korsgaard, Jonas Blomberg, Christina Welinder-Olsson, Björn Herrmann

**Affiliations:** 1 Section of Clinical Bacteriology, Department of Medical Sciences, Uppsala University, Uppsala, Sweden; 2 Department of Infectious Diseases, Örebro University Hospital, Örebro, Sweden; 3 Department of Chest Diseases, Aarhus University Hospital, Aalborg, Denmark; 4 Section of Clinical Virology, Department of Medical Sciences, Uppsala University, Uppsala, Sweden; 5 Department of Clinical Bacteriology, Sahlgrenska University Hospital, Gothenburg, Sweden

## Abstract

**Background:**

*Streptococcus pneumoniae *and *Haemophilus influenzae *cause pneumonia and as *Neisseria meningitidis *they are important agents of meningitis. Although several PCR methods have been described for these bacteria the specificity is an underestimated problem. Here we present a quantitative multiplex real-time PCR (qmPCR) for detection of *S. pneumoniae *(9802 gene fragment), *H. influenzae *(*omp P6 *gene) and *N. meningitidis (ctrA *gene). The method was evaluated on bronchoalveolar lavage (BAL) samples from 156 adults with lower respiratory tract infection (LRTI) and 31 controls, and on 87 cerebrospinal fluid (CSF) samples from meningitis patients.

**Results:**

The analytical sensitivity was not affected by using a combined mixture of reagents and a combined DNA standard (*S. pneumoniae/H. influenzae/N. meningitidis*) in single tubes. By blood- and BAL-culture and *S. pneumoniae *urinary antigen test, *S. pneumoniae *and *H. influenzae *were aetiological agents in 21 and 31 of the LTRI patients, respectively. These pathogens were identified by qmPCR in 52 and 72 of the cases, respectively, yielding sensitivities and specificities of 95% and 75% for *S. pneumoniae*, and 90% and 65% for *H. influenzae*, respectively. When using a cut-off of 10^5 ^genome copies/mL for clinical positivity the sensitivities and specificities were 90% and 80% for *S. pneumoniae*, and 81% and 85% for *H. influenzae*, respectively. Of 44 culture negative but qmPCR positive for *H. influenzae*, 41 were confirmed by *fucK *PCR as *H. influenzae*. Of the 103 patients who had taken antibiotics prior to sampling, *S. pneumoniae *and *H. influenzae *were identified by culture in 6% and 20% of the cases, respectively, and by the qmPCR in 36% and 53% of the cases, respectively.

In 87 CSF samples *S. pneumoniae *and *N. meningitidis *were identified by culture and/or *16 S rRNA *in 14 and 10 samples and by qmPCR in 14 and 10 samples, respectively, giving a sensitivity of 100% and a specificity of 100% for both bacteria.

**Conclusions:**

The PCR provides increased sensitivity and the multiplex format facilitates diagnosis of *S. pneumoniae*, *H. influenzae *and *N. meningitidis *and the assay enable detection after antibiotic treatment has been installed. Quantification increases the specificity of the etiology for pneumonia.

## Background

*Streptococcus pneumoniae *and *Haemophilus influenzae *are major causes of community-acquired pneumonia (CAP) [1,2] and as *Neisseria meningitidis *they are important agents of meningitis [3-5]. Identification of the microbiological cause of CAP and meningitis is important, as it enables pathogen-directed antibiotic therapy. Conventional detection of bacteria is based on culture and phenotypic characterization. However, culture methods are time-consuming and have relatively low sensitivity, especially when antibiotics have been given to the patient prior to sampling [6]. The use of nucleic acid amplification tests, such as quantitative real-time polymerase chain reaction (qPCR), have enabled more sensitive and rapid detection of pathogens in respiratory secretions and cerebrospinal fluid (CSF).

Several qPCR assays for the detection of *S. pneumoniae *[7-9], *H. influenzae *[10-12] and *N. meningitidis *[13] have been developed and multiplex detection of several target DNAs in a single tube is achievable [14-16]. Still, the specificity of methods used is an underestimated problem and commonly used targets have been shown to be unspecific and causing misleading results. An illustrative example is the pneumolysin (*ply*) gene for the detection of *S. pneumoniae *[17-19]. For detection of *H. influenzae*, a species with frequent exchange of genetic elements, the problem is even worse and most target genes used are problematic. The *bexA *is not present in all strains of *H. influenzae *[20], while *16 S rRNA *and *rnpB *do not provide specific detection [21]. We have recently developed qPCRs for specific detection of *S. pneumoniae*, based on the Spn9802 fragment [17], and for the detection of *H. influenzae*, based on the outer membrane protein *P6 *[21]. Real time PCR assays for detection of *N. meningitidis *have been based on genes as *porA *[22] and *ctrA *[14,16].

Here we present a new quantitative multiplex PCR (qmPCR) method for detection of *S. pneumoniae*, *H. influenzae *and *N. meningitidis*. The method was evaluated on a collection of bronchoalveolar lavage (BAL) and cerebrospinal fluid specimens for detection of lower respiratory tract infection (LRTI) and meningitis due to these three bacteria species.

## Methods

### Patients and controls

From 1997 through 2000, 159 consecutively identified immunocompetent adult patients who were hospitalised for LRTI at the Department of Internal Medicine, Silkeborg County Hospital, Silkeborg, Denmark, were enrolled in a prospective study [23]. The criteria for LRTI were fever and/or an increased leukocyte count (≥ 11 × 10^9 ^/L), together with increased focal symptoms from the lower airways with at least one of three newly developed symptoms of increased dyspnoea, increased coughing and/or increased sputum purulence.

The enrolled patients underwent standardized fibre-optic bronchoscopy within 24 hours from admission. For the present study, BAL fluid was available in 156 patients, median age 63 years (range 26-90 years). A chronic lung disease was documented in 72 patients (46%), 31% were current and 40% were previous smokers. New X-ray infiltrates were identified in 87 patients (56%). Antibiotics had been taken within 7 days prior to bronchoscopy in 103 cases (66%).

As controls, 31 adult patients, median age 64 years (range 30-77 years), who consecutively underwent fibre-optic bronchoscopy for suspected malignancy and who did not have pulmonary infection were included. Nineteen of them had lung malignancies and 12 had no pathology identified by bronchoscopy or radiological examinations. Twenty-seven controls (87%) were current or previous smokers.

CSF samples sent for culture to the Bacteriological Laboratory, Sahlgrenska University Hospital, Gothenburg, Sweden during a four year period were used in the study. Specimens were eligible if the total CSF white blood cell (WBC) count was ≥10 × 10^6 ^/L indicating meningeal inflammation. Only one CSF sample from each patient was included. Medical records of all patients included in the study were reviewed retrospectively for a final diagnosis, predisposing factors, treatment and outcome by one doctor. All 87 specimens were included in a study previously published for *16 S rRNA *gene PCR [24] and the relevance of the PCR findings and bacterial cultures to the final diagnosis was evaluated and compared with the clinical findings and other laboratory results. The median age of the patients were 34 years (range 1 day- 91 years).

### Fibre-optic bronchoscope

In brief, the fibre-optic bronchoscope was introduced through the nose or through the mouth. The tip of the bronchoscope was wedged into the segment of bronchus affected by a pulmonary infiltrate, or, if no infiltrate was available, into the middle lobe. A sterile, thin tube was then introduced into the working channel of the bronchoscope, and lavage was then performed. One to three portions of 60 mL of isotonic NaCl were used for lavage, and the aspirated fluid was collected in one single portion for microbiological analyses.

### Conventional diagnostic methods

BAL fluid from the LRTI patients and the controls were analysed with culture (but no Gram staining) at the Department of Clinical Microbiology, Aarhus University Hospital (Aalborg, Denmark), within a maximum of 6 h from the time of sampling. The specimens were cultured on 5% horse blood agar and chocolate agar with semi-quantitative determinations by dispersion of 1 and 10 μL on each half of the plate. The plates were incubated in 5% carbon dioxide at 35°C for 24-48 h.

From 152 LRTI patients, blood samples were collected for culture with a Bactec blood-culturing system (BioMérieux, Marcy-Etoile, France) at the Department of Clinical Microbiology, Aarhus University Hospital. Non-frozen urine samples collected from 142 LRTI patients were sent to the Department of Bacteriology, Mycology and Parasitology, Statens Serum Institute, Copenhagen, Denmark, and were analyzed for pneumococcal capsular polysaccharides by countercurrent immunoelectrophoresis [25].

CSF samples were submitted for routine bacterial culture and chemistry [26].

### DNA extraction

DNA from 0.2-0.5 mL BAL was extracted by the automatic MagNa Pure LC DNA-Isolation system (Roche Diagnostics). Bacteria DNA used for determination of the analytical sensitivity of the Spn9802 and the *P6 *PCRs was purified from cultured isolates (*S. pneumoniae *CCUG 28588^T ^and *H. influenzae *CCUG 23946 ^T^) by phenol-chloroform extraction of bacteria harvested in exponential growth phase after culturing on chocolate agar at 37°C in 5% carbon dioxide and the concentration of DNA was determined by a Nanodrop instrument (NanoDrop Technologies, Inc. Wilmington, DE, USA). The genome copy number was determined according to conventional calculations based on molecular weight and one gene copy per genome.

CSF samples (50 μL-1.5 mL) were centrifuged at 12 000 g for 20 min, after which DNA was extracted from the pellet with a bacterial DNA preparation kit (Roche Diagnostics, Indianapolis, USA), used according to the manufacturer's instructions.

### qmPCR

The quantitative Spn9802 PCR for the detection of *S. pneumoniae *[17] was combined with the *P6 *PCR for the detection of *H. influenzae *[21] and the *ctrA *PCR for the detection of *Neisseria meningitidis *[14]. All primers and probes are shown in Table 1 where positions with lower case letters indicate locked nucleic acid [27].

**Table 1:** Oligonucleotide primers and probes for detection of S. pneumoniae, H. influenzae and N. meningitidis.

	Sequence (5' to 3')^a^	Positions in target gene
*S. pneumoniae*		
Spn9802 F	5'-A GTC GTT CCA AGG TAA CAA GTC T-3'	3370-3392
Spn9802 R	5'-AC CAA CTC GAC CAC CTC TTT-3'	3525-3506
Spn9802 FAM	5'-^FAM^-aTc AGa TTg CTg ATa AAa CgA-^BHQ1^-'3	
		
*H. influenzae*		
Hi P6 F	5'-CCA GCT GCT AAA GTA TTA GTA GAA G-3'	302-326
Hi P6 R	5'-TTC ACC GTA AGA TAC TGT GCC-3'	477-457
Hi P6 JOE	5'-^JOE^- CAg ATg CAg TTg AAg GTt Att tAG-^BHQ1^-'3	
		
*N. meningitidis*		
ctrA F	5'-GCTGCGGTAGGTGGTTCAA-3'	617-635
ctrA R	5'-TTGTCGCGGATTTGCAACTA-3'	727-708
ctrA ROX	5'-^ROX^-CATTGCCACGTGTCAGCTGCACAT- ^BHQ1^-'3	

^a ^Positions with lower case letters indicate locked nucleic acid [27].

The PCR for detection of *N. meningitidis *was used as described previously, except that 3.5 mmol/L MgCl_2 _was used instead of 5.5 mmol/L and that the elongation time was 40 s instead of 1 min. All primers and probes were obtained from Thermo Hybaid, Interactiva Division (Ulm, Germany) except the Spn9802 FAM probe which was obtained from Eurogentec, Seraing, Belgium.

The real-time PCR assay was performed in a Rotor-Gene 3000 instrument (Qiagen, Hilden, Germany). The optimized real-time PCR amplifications were performed in 25-μL reactions containing 0.3 μmol/L of each primer, 0.2 μmol/L of the Spn9802 FAM probe, 0.1μmol/L of the P6 JOE probe and ctrA ROX probe, 3.5 mmol/L MgCl_2_, 0.2 mmol/L deoxynucleoside triphosphate, and 1 U HotStar Taq polymerase (Qiagen) in PCR buffer. A total of 5 μL of target DNA was used in the assay. The qmPCR was performed according to the following program: 15 min of enzyme activation at 95°C, followed by 45 cycles of 95°C for 15 s and 60°C for 40 s.

### Reproducibility of analytical sensitivity and quantification

The analytical sensitivity of the Spn9802, *P6 *and *ctrA *PCRs was determined by serial dilutions of target DNA in carrier tRNA (1μg/mL). Two experiments were performed with 5 to 600 genome copies per reaction tube and 2 to 4 tubes of each dilution.

The reproducibility of quantification was evaluated by testing DNA preparations with known concentrations (duplicates of 500, 2,000 and 10,000 genome copies per PCR reaction) in five consecutive runs and also in 73 BAL samples and in 8 CSF samples. PCRs with primer/probe reagents in both monoplex and multiplex were tested in parallel. In addition we tested the reproducibility of quantification with positive control DNA of *S. pneumoniae*, *H. influenzae *and *N. meningitidis *in separate tubes and combined in a single tube.

### *fucK *PCR

The *fucK *PCR was used as previously described [28], to confirm the presence of *H. influenzae *in samples which proved negative by culture but positive by qmPCR.

### *lytA *PCR

For respiratory samples the *lytA *PCR was tested in a gel based PCR for *S. pneumoniae *as previously described [29]. In short, extracted DNA (10 μL) was added to a PCR mixture, and after 40 cycles, PCR products were detected on ethidium bromide-stained agarose gels. By serial dilution of bacterial strains, the detection level of *lytA *PCR has been shown to be 10^2 ^colony forming units (CFU)/mL sample [29].

### *16 S rRNA *PCR for CSF samples

The primers and other PCR conditions used to amplify the 5'-half of the *16 S rRNA *gene were previously described [24]. The PCR product was visualized in an agarosegel and DNA bands of expected size were cut from the gel, purified with a Qiaquick Gel Extraction kit (Qiagen) and subjected to cycle sequencing using the ABI prism Big Dye Terminator Sequencing Ready Reaction kit, v.1.1 (Applied Biosystems). The sequencing reaction products were analyzed using an ABI PRISM 310 Genetic Analyser (Applied Biosystems). After DNA sequence editing, the GenBank BLAST program was used for sequence comparisons.

### Ethics

The study was performed according to the Declaration of Helsinki II and approved by the local ethical committee and all participating patients gave written consent.

## Results

A sensitive and specific multiplex PCR for quantitative detection of *S. pneumoniae, H. influenzae *and *N. meningitidis *was developed and evaluated on BAL samples from adults with LRTI and a control group, and on CSF samples from patients with meningitis. To establish the detection capacity of the Spn9802, the *P6 *and the *ctrA *assays, serial dilutions of target DNA with known concentration were repeatedly tested and the analytical sensitivity was 10-60 copies per PCR reaction for the Spn9802 assay, 3-30 copies per PCR reaction for the *P6 *assay and 5-50 copies per PCR reaction for the *ctrA *assay. As shown in Table 2 the analytical sensitivity and quantification was not affected by using a combined mixture of reagents and a combined DNA standard (*S. pneumoniae, H. influenzae *and *N. meningitidis*) in single tubes.

**Table 2:** Detection capacity of multiplex quantitative PCR.

Oligos for a single target			Oligos for three targets			Δ *Ct*	Δ copy number (log 10)
**DNA standard copy number** **of target DNA (number of reactions)**	**Mean *Ct *value**	**Mean measured copy** **number (log10)**	**DNA standard *S. pneumoniae,*** ***H. influenzae ***and ***N. meningitidis*** **copy number of each target DNA**	**Mean *Ct *value**	**Mean measured copy number (log10)**		

Spn 10000 (5)	27.7			27.8		0.1	
Spn 2000 (5)	30.2			30.4		0.2	
Spn 500 (7)	32.7			32.4		-0.3	
							
Hi 10000 (5)	23.8			23.7		-0.1	
Hi 2000 (5)	26.4			26.4		0.0	
Hi 500 (7)	28.6			28.5		-0.1	
							
Mc 10000 (4)	27.6			27.4		-0.2	
Mc 2000 (4)	30.5			30.0		-0.5	
Mc 500 (6)	32.5			32.3		-0.3	
							
Spn (23 clinical samples)	27.7 ± 7.6	3.9 ± 1.8		28.2 ± 7.6	3.8 ± 2.0	0.5	-0.1
Hi (50 clinical samples)	24.1 ± 10.7	3.9 ± 2.8		24.7 ± 7.6	3.8 ± 3.0	0.6	-0.1
Mc (8 clinical samples)	22.0 ± 1.9	5.2 ± 0.5		22.2 ± 2.0	5.2 ± 0.5	0.2	0

*Ct *= Cycle of threshold; Spn = *S. pneumoniae*; Hi = *H. influenzae*; Mc = *N. meningitidis*

Comparison of using PCR reaction mix with a single DNA standard and oligos for one target organism versus triplex DNA target standard and oligos for 3 target organisms.

Table 3A shows results of tests for *S. pneumoniae *and *H. influenzae *in the patient group. Of 156 LRTI patients *S. pneumoniae *was identified by conventional tests in 21 (13%) cases, and by qmPCR in 54 (35%) cases, including 47 cases using a cut-off level of 10^5 ^copies/mL.

**Table 3:** Comparison of reference tests with quantitative multiplex PCR (qmPCR).

Results			
		
**Reference tests **^a^	qmPCR^b^	No. of patients	No. on antibiotic treatment
**A**.			
Spn & Hi	Spn & Hi	1	1
Spn & Hi	Hi	1	1
Spn	Spn & Hi	5	4
Spn	Spn	14	6
-	Spn	20	15
-	Spn & Hi	9	7
Hi	Spn & Hi	5	5
Hi	Hi	21	12
Hi	-	3	3
-	Hi	30	26
-	-	47	24
**B**.			
Spn	Hi	1	
Spn	Spn	1	
Hi	Spn & Hi	1	
Hi	Hi	2	1
-	Spn	3	1
-	Spn & Hi	3	
-	Hi	4	
-	-	16	1

^a ^Blood culture, urinary antigen test, and BAL culture for *S. pneumoniae*; blood culture and BAL culture for *H. influenzae*.; -, negative test result.

^b ^Spn9802+ was considered as *S. pneumoniae*+; P6+ was considered as *H. influenzae*+.

Results of reference tests and qmPCR for *Streptococcus pneumoniae *(Spn) and *Haemophilus influenzae *(Hi) applied to bronchoalveolar lavage (BAL) samples in 156 patients with lower respiratory tract infection (A) and in 31 control patients (B).

From the 21 patients with conventional (blood culture, BAL culture, or urinary antigen test) tests positive for *S. pneumoniae*, 20 were positive by qmPCR. In addition 34 cases with no conventional test positive for *S. pneumoniae *were positive with Spn9802 PCR of which 26 were also positive by *lytA *PCR. Of the 6 patients with pneumococcal bacteraemia, *S. pneumoniae *was identified by BAL culture in one case, by urinary antigen test in one case, and by qmPCR and *lytA *PCR in all the 6 patients. Similarly, among the 9 patients with positive urinary antigen test, *S. pneumoniae *was identified in 8 by BAL qmPCR and in seven by *lytA *PCR, and none by BAL culture.

*H. influenzae *was not found in any blood culture but was detected by BAL culture in 31 cases, of which 28 also were positive by qmPCR. Of 44 cases proved negative by culture but positive by qmPCR, 41 were confirmed by *fucK *PCR.

Among the 31 control patients *S. pneumoniae *and *H. influenzae *were identified by BAL culture in 2 (6%) and 3 (10%) cases respectively, by qmPCR in 8 (26%) and 11 (35%) cases (Table 3B). Of 7 and 8 cases proved negative by culture but positive with qmPCR for *S. pneumoniae *and *H. influenzae *respectively, 2 were positive by *lytA *PCR for *S. pneumoniae *and 7 were positive by *fucK *PCR for *H. influenzae*.

**Figure 1:**
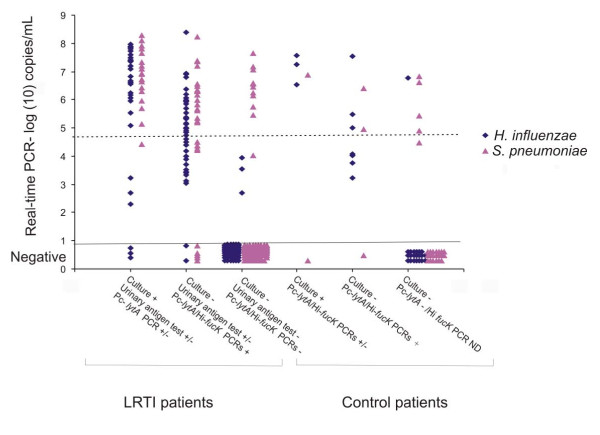
**Multiplex real-time PCR copy numbers of target organisms in patients and controls**. Comparison of PCR copy numbers in the LRTI patients and controls compared with culture, urinary antigen test and gel-based *lytA *PCR.

Figure 1 shows the qmPCR copy number of the LRTI patients and controls compared to results by culture, urinary antigen test and *lytA *PCR. Among the qmPCR positive subjects, the LRTI patients and controls had a similar mean log 10 of copy number 5.69 (standard deviation [SD] 1.53) versus 5.65 (SD 1.63); *p *= 0.79, for *H. influenzae *and 6.31 (SD 1.12) versus 5.93 (SD 0.96); *p *= 0.36, for *S. pneumoniae*). If the cut-off limit for a positive qmPCR result was risen to 10^5 ^DNA copies/mL, the positivity rate among the controls would drop from 26% (8/31) to 16% (5/31) for *S. pneumoniae *and from 35% (11/31) to 19% (6/31) for *H. influenzae*. Similarly in the patient group the positivity rate would drop from 35% (54/156) to 30% (47/156) for *S. pneumoniae *and from 46% (72/156) to 20% (31/156) for *H. influenzae*.

Table 4 shows the sensitivities and specificities of the qmPCR, with the detection limit of the PCR assay itself and a detection limit of 10^5 ^copies/mL. Since one urinary antigen test positive patient had Spn9802 DNA determined at <10^5 ^copies/mL and three culture positive patients had *P6 *DNA determined at <10^5 ^copies/mL, the raise of the cut-off limit to 10^5 ^copies/mL, would drop the sensitivities, but the specificities would increase.

**Table 4:** Sensitivities and specificities of multiplex real-time PCR for detection of S. pneumoniae and H. influenzae.

Species	Reference test	Detection limit of the assay				Cutoff 10^5 ^copies/mL			
		
		Sensitivity	Specificity	PPV^a^	NPV^b^	Sensitivity	Specificity	PPV	NPV
*S. pneumoniae*	BAL culture, blood culture and urinary antigen test	95% (20/21)	75% (101/135)	37% (20/54)	99% (101/102)	90% (19/21)	80% (108/135)	41% (19/46)	98% (108/110)
	BAL culture, blood culture and urinary antigen tes + *lytA *PCR	91% (43/47)	89% (97/109)	78% (43/55)	96% (97/101)	79% (37/47)	95% (104/109)	88% (37/42)	91% (104/114)
*H. influenzae*	BAL culture^c^	90% (28/31)	65% (81/125)	39% (28/72)	96% (81/84)	81% (25/31)	85% (106/125)	57% (25/44)	95% (106/112)
	BAL culture^c ^+ *fucK *PCR^d^	93% (69/74)	96% (79/82)	96% (69/72)	94% (79/84)	63% (47/74)	100.0% (82/82)	100% (47/47)	75% (82/109)

^a ^Positive predictive value

^b ^Negative predictive value

^c ^Blood culture were also performed for H. influenzae but all were negative

^d ^*fucK *PCR was performed in the PCR positive and culture negative samples

Analysis of bronchoalveolar lavage from 156 adults with lower respiratory tract infection.

Among 103 patients treated with antibiotic before sampling, *S. pneumoniae *and *H. influenzae *were identified by culture in 6% (6/103) and 20% (21/103) respectively, and by qmPCR in 36% (37/103) and 53% (55/103) respectively. Of 22 patients positive by Spn9802 PCR and *lytA *PCR alone 19 of them had antibiotics prior to sampling.

**Figure 2:**
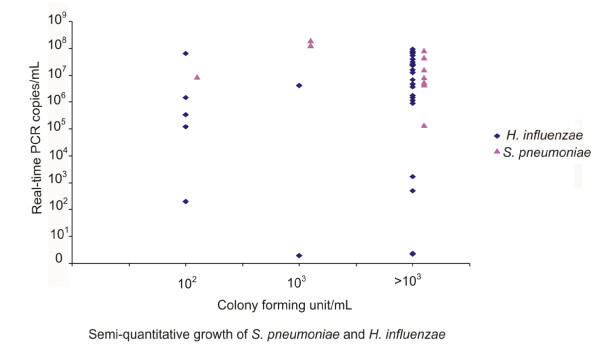
**Quantitative results of the multiplex real-time PCR compared to semi-quantitative culture of BAL specimens**.

Figure 2 shows the quantitative results of the qmPCR compared to semi-quantitative culture of BAL specimens for *S. pneumoniae *and *H. influenzae*. There was no correlation between the measured DNA copy number/mL and the bacterial growth.

Table 5 shows results of tests for *S. pneumoniae *and *N. meningitidis *in patients with meningitis. Of 87 CSF samples, *S. pneumoniae *and *N. meningitidis *were detected by culture in 5 (6%) and 2 (2%) samples, by *16 S rRNA *PCR in 14 (16%) and 10 (11%) and by qmPCR and in 14 (16%) and 10 (11%) samples respectively. Altogether, culture, *16 S rRNA *PCR and qmPCR were positive for *S. pneumoniae *in 14 cases, *N. meningitidis *in 10 cases, and *H. influenzae *in no case. If culture and the *16 S rRNA *PCR in combination were used as reference standard for aetiology of meningitis, the sensitivities and specificities would be 100% and 100% for both *S. pneumoniae *and *N. meningitidis*. Two samples positive by the *ctrA *PCR were positive in the unspecific *16 S rRNA *PCR and sequence analysis of the PCR product determined them as *Neisseria *spp. They were considered as *N. meningitidis *in the specificity calculation. Of 17 samples that were negative by culture but positive by qmPCR 8 samples originated from patients that were under antibiotic treatment (Amoxyclav PO, Cefotaxime IV or Cefuroxime IV) before lumbar puncture.

**Table 5:** Results of tests for S. pneumoniae and N. meningitidis in 87 patients with meningitis.

Bacterial species	Culture and/or microscopic examination	*16 S rRNA *PCR	qmPCR		Total number	No. on antibiotic treatment
					
			Spn9802 PCR	*ctrA *PCR		
*S. pneumoniae*	+	+	+		5	2
	-	+	+		9	5
						
*N. meningitidis*	+	+		+	2	
	-	+ ^a^		+	8	3
	- ^b^	-	-	-	63	

^a ^*Neisseria *spp DNA was detected by *16 S rRNA *PCR in 2 samples and sequence determined as *Neisseria spp*. Here considered as *N. meningitidis*

^b ^Negative for *N. meningitidis *and *H. influenzae*

## Discussion

In this study we established a sensitive detection system that enabled simultaneous quantification of *S. pneumoniae, H. influenzae *and *N. meningitidis *DNA using qmPCR. The multiplex assay was reproducible and no change in detection and quantification capacity was seen when a combined mixture of reagents and a combined DNA standard (*S. pneumoniae/H. influenzae/N. meningitidis*) in single tubes was used (Table 2). This multiplex PCR assay reduced the expense of reagents and the required time for analysis.

Antibiotic treatment prior sampling has been found to reduce the positivity rate of BAL culture from 92% to 55% in patients with severe community acquired pneumonia [6]. In this study 66% (103/156) of the patients had antibiotic therapy prior the sampling, this high rate of antibiotic treatment is probably the reason for the suboptimal specificity of the qmPCR. Of 78 samples which were negative by culture and positive for *S. pneumoniae *or *H. influenzae *by the qmPCR, 64 were treated with antibiotic 0-7 days prior to sampling. The high rate of prior antibiotic treatment was probably also the reason for the lack of correlation between DNA concentration and bacterial concentration determined by semi-quantitative culture (Figure 2). This lack of correlation between quantification of target DNA and culture contrasts to our previous analysis of nasopharyngeal aspirates from community acquired pneumonia patients, where a significant correlation was seen, but only 25% of patients were on antibiotic treatment when samples were collected in the previous study [17,21].

The evaluation of nucleic acid amplification tests by comparison with less sensitive reference methods such as culture is problematic. Several imperfect tests may be used to define a composite reference standard [30]. An alternative way to resolve cases with different test results is to use discrepant analysis where an additional method is used to determine the specimen status. Such analyses have been criticized [31], but is often the most realistic procedure for the evaluation of new methods that are more sensitive than an established reference method. In our study the Spn9802 target was evaluated by a composite reference standard and for the *P6 *target discrepant analysis was used. This resulted in increased specificity and a higher number of pneumonia cases with defined etiology. As expected the positive predictive values increase with increased specificity, thus with a cut-off limit of 10^5 ^copies/mL a positive PCR result is very reliable using an extended reference standard.

We have recently shown that Spn9802 PCR and *P6 *PCR are specific for *S. pneumoniae *and *H. influenzae *when bacterial strains have been tested.

Nevertheless, colonization of *S. pneumoniae *and *H. influenzae *in the respiratory tract is problematic for both culture and PCR. To overcome this problem semi-quantitative culture is often used. In our study a detection limit of 10^5 ^DNA copies/mL for positive Spn9802 and *P6 *PCRs yielded a high specificity but somewhat reduced the sensitivity. Similar results have been seen in previous studies [6,32,33] based on BAL culture and demonstrated that a cut-off of 10^4^-10^5 ^CFU/mL allow differentiation between colonization and infection of the lower respiratory tract. However, CFU/mL does not automatically correspond to the number of DNA copies/mL since several bacteria may aggregate and generate one colony although they constitute several genome equivalents. Furthermore, as described above antibiotic treatment before sampling and smoking habits have an effect on the number of detected bacteria. Thus patient treatment and the patient group characteristics affect the possibility of using quantification to differentiate between colonization and infection.

When the multiplex PCR was applied on CSF samples, our assay was able to detect all the cases of *N. meningitidis *and *S. pneumoniae *that were found by culture and/or 16 S PCR in a previous study [24]. The problem of choosing optimal targets for *S. pneumonia *and *H. influenzae *has been addressed above. The primer pair used for *N. meningitidis *in our assay has previously been used in a multiplex assay for detection of bacterial meningitis [14] and even been evaluated in a major interlaboratory comparison of PCR-based identification of meningococci [34] as well as in other studies with satisfying results [35,36].

## Conclusions

Although culture is still indispensable in bacteriological diagnostics multiplex PCR enables concurrent diagnostics of viruses and fungi and provides a powerful tool for analysis. We conclude that the multiplex format of the assay facilitates diagnostics of *S. pneumoniae*, *H. influenzae *and *N. meningitidis *and is suitable for analysis of both respiratory tract tract and CSF specimens. The assay also enable detection after antibiotic treatment has been installed. Quantification increases the specificity of etiology for pneumonia.

## Authors' contributions

GA: BH, KS and JB have planned the study; GA has done the laboratory work and written the draft. KS, JK and CW have provided clinical materials. All authors have contributed intellectually during the writing process and have read and approved the final manuscript.
